# Research progress on the role of CD14 in osteoarthritis

**DOI:** 10.3389/fimmu.2026.1824403

**Published:** 2026-07-28

**Authors:** Lei Yang, Shuxing Xing

**Affiliations:** Department of Orthopedics, Chengdu Fifth People’s Hospital, Chengdu, China

**Keywords:** CD14, inflammation, macrophages, osteoarthritis, targeted therapy

## Abstract

Osteoarthritis (OA) is a complex degenerative disease centered on inflammation and involving multi−cellular cooperation, with a continuously rising global burden. Current therapeutic measures mainly focus on symptom relief, slowing progression, and end−stage joint arthroplasty; no disease−modifying or reversal agent is yet available, underscoring the urgent need to dissect the inflammatory driving mechanisms and identify effective intervention targets. As a pattern recognition receptor, CD14 is highly expressed on synovial macrophages in the OA microenvironment. It recognizes endogenous damage−associated molecular patterns, driving pro−inflammatory responses through both TLR4−dependent (MyD88/NF−κB) and TLR4−independent (NLRP3 inflammasome) pathways. Recent studies have accumulated substantial evidence; clinical samples and animal models consistently show that CD14 deficiency or blockade reduces cartilage damage, synovitis, and pain, while soluble CD14 (sCD14) levels positively correlate with inflammatory cytokines and clinical symptoms, indicating its important role in OA pathogenesis and supporting its value as a biomarker and therapeutic target. This review aims to integrate current evidence, clarify the central pro−inflammatory role of CD14 in OA, and provide a theoretical basis for future precision targeted therapies and biomarker−driven clinical trial designs, thereby advancing the development of diagnostic and therapeutic strategies for OA.

## 1 Introduction

Osteoarthritis (OA) is the leading cause of pain, activity limitation, and disability in middle−aged and elderly populations, and its global disease burden continues to escalate (1–3). The Global Burden of Disease (GBD) study has shown that over the past three decades, the prevalence, incidence, and years lived with disability due to OA have all increased significantly. Currently, approximately 240 million people worldwide are affected, with a prevalence of about 12% among those aged ≥60 years, and among community−dwelling individuals over 65 years, the proportion of symptomatic knee or hip OA can reach as high as 40% (4, 5). With population aging and rising obesity rates, the burden of OA is extending into the working−age population aged 30–44 years (6).

The epidemiology of OA exhibits significant age− and sex−related heterogeneity. Prevalence rises sharply with age; approximately 70% of individuals over 70 years have radiological or clinical manifestations (7). Women not only have higher prevalence (knee OA at age ≥60: 13% in women vs. 10% in men) and disability risk than men, but also experience more severe pain and worse functional outcomes. Moreover, marked differences in diagnosed prevalence among EU countries (from 2.8% in Romania to 18.3% in Hungary) suggest that regional and sociodemographic index (SDI) factors profoundly influence disease distribution (8, 9). Current clinical treatment remains symptom−oriented (non−steroidal anti−inflammatory drugs, intra−articular injections, and end−stage joint replacement), and no disease−modifying osteoarthritis drug (DMOAD) capable of slowing, halting, or reversing structural damage has received regulatory approval from agencies such as the FDA. Numerous drug candidates have failed to achieve clinical translation due to insufficient efficacy, difficulties in delivery to the joint microenvironment, or lack of ideal targets. Therefore, in−depth elucidation of OA pathogenesis and identification of effective intervention targets have become critical scientific issues to address urgent clinical needs and reduce the public health burden (10–13).

Recent studies have established that OA is not merely a degenerative process but a complex network centered on inflammation, involving multi−cellular cooperation and multi−pathway crosstalk (14–16). Inflammation not only directly drives cartilage degradation and synovial pathology but also interacts closely with cellular senescence, dysregulated signaling pathways, and remodeling of the immune microenvironment, forming a self−perpetuating pathological cycle. It is currently believed that endogenous danger signals—damage−associated molecular patterns (DAMPs)—released after joint tissue injury activate the innate immune system, triggering persistent low−grade inflammation, which in turn promotes synovitis, cartilage degradation, subchondral bone remodeling, and pain sensitization (17–19).

In the OA joint microenvironment, various immune cells (especially macrophages) are activated by DAMPs and become major sources of inflammatory cytokines and proteases. CD14−positive macrophages are markedly enriched in synovial tissue, with significantly higher TNF−α and IL−1β mRNA expression levels than CD14^-^ cells; *in vitro* experiments further confirmed that IL−1β and TNF−α can induce synovial cells and fibroblasts to express nerve growth factor (NGF), contributing to pain generation (20). Moreover, in a post−traumatic OA equine model, the number of CD14^+^ macrophages in synovial fluid was significantly increased, accompanied by a higher proportion of Th17 cells, suggesting that CD14^+^ immune cells together with DAMP signaling pathways participate in the inflammatory cascade of OA (21). These results emphasize the importance of immune cells recognizing DAMPs to initiate and sustain local joint inflammation.

CD14, as a key pattern recognition receptor, has long been considered to play a central role in bacterial lipopolysaccharide (LPS) recognition; recent studies have revealed that it can also recognize multiple endogenous DAMPs (e.g., HMGB1, mitochondrial DNA), thereby mediating sterile inflammatory responses. In OA, CD14 is highly expressed in synovial tissue and is significantly enriched in inflammation−related pathways such as the TLR4 signaling pathway, cytokine−cytokine receptor interaction, and the MAPK pathway (22). Clinical sample analyses show that soluble CD14 (sCD14) levels in OA synovial fluid are positively correlated with IL−6, IL−1β, TNF−α, and complement C3, and the proportion of CD14^+^ monocytes is elevated in recurrent cases; *in vitro* experiments confirmed that recombinant CD14 together with LPS co−stimulation significantly promotes the secretion of IL−6, IL−8, and MMP−3 by fibroblast−like synoviocytes (23). More importantly, in mouse post−traumatic OA models, CD14 gene deficiency or local blockade significantly alleviates cartilage damage, synovial inflammation, pain behaviors, and motor dysfunction. These lines of evidence collectively point to a critical role of CD14 in the inflammatory driving mechanism of OA (24, 25). However, the specific mechanisms of CD14 in OA, the functional differences among its subtypes, and its translational potential as a biomarker or therapeutic target still require systematic synthesis. Therefore, this review aims to integrate existing studies and comprehensively discuss the molecular characteristics, signaling mechanisms, pathological functions, and clinical translation prospects of CD14 in OA, providing a theoretical foundation for the development of novel immunomodulatory strategies.

## 2 Molecular characteristics and expression profile of CD14

CD14 is a highly conserved glycosylphosphatidylinositol (GPI)−anchored membrane protein encoded by the CD14 gene, located on human chromosome 5q31.1. This molecule lacks a transmembrane domain and an intracellular signaling domain, and it primarily mediates signal transduction through cooperation with co−receptors such as Toll−like receptors (TLRs). The CD14 protein is rich in leucine−rich repeats (LRRs), which constitute its ligand−recognition domain and can specifically bind various pathogen−associated molecular patterns (PAMPs) and DAMPs. In synovial tissue from OA patients, the CD14 gene is highly expressed and has been identified as a pivotal hub gene in inflammation−related pathways, with its expression significantly enriched in the Toll−like receptor signaling pathway, TNF signaling pathway, and cytokine−cytokine receptor interaction networks (22, 26, 27).

CD14 exists in two major forms: membrane−bound CD14 (mCD14) and soluble CD14 (sCD14). mCD14 is anchored via GPI on the surface of myeloid cells (e.g., monocytes/macrophages) and serves as a co−receptor for ligands such as LPS to participate in TLR4 signal activation; The main source of sCD14 is derived from CD14, which is expressed on the membranes of myeloid cells such as monocytes and macrophages. It is released after the activation of cells and undergoes serine protease-dependent protein hydrolytic cleavage (28–30).

CD14 is mainly expressed on myeloid cells, especially classical monocytes (CD14^++^CD16^-^), intermediate monocytes (CD14^++^CD16^+^), and tissue−resident macrophages. Under inflammatory conditions, different monocyte subsets exhibit differential responses to stimulation: intermediate monocytes show the most pronounced upregulation of CD11b and increases in IL−6 and IL−8 mRNA after LPS challenge, suggesting their dominant role in inflammatory responses (31). In addition, CD14 can also be detected in some dendritic cell precursors, perivascular cells in synovial tissue, and certain T−cell subsets (32, 33). For example, in the synovium of knee OA patients, CD8^+^ T cells show a trend toward increased CD14 expression; in rheumatoid arthritis synovium, CD14^+^ cells can exhibit dendritic morphology and express CD90, with the potential to differentiate into dendritic cells (34). These findings indicate that the cellular distribution of CD14 is highly heterogeneous and may dynamically change with disease status.

In the OA joint microenvironment, CD14 is primarily enriched in myeloid cells within synovial tissue and synovial fluid. Studies show that the number of CD14^+^ macrophages in synovial membrane monocytes from knee OA patients is four−fold higher than that in hip OA patients (32). In the equine post−traumatic OA model, the number of CD14−positive macrophages in synovial fluid was significantly higher at the moderate disease stage than in mild and control groups (21). In human OA synovial tissue, CD14−positive areas largely correspond to macrophage−rich regions, where TNF−α and IL−1β mRNA levels are significantly higher than in CD14−negative (fibroblast−rich) areas (20). Furthermore, flow cytometry analysis confirmed that the proportion of CD14^+^CD16^-^ monocytes in OA synovial fluid was significantly higher in recurrent patients than in first−episode patients, and this subset highly expressed TLR4 (23). These data collectively demonstrate that CD14^+^ myeloid cells extensively infiltrate the local OA joint and are in a highly activated state, constituting a critical component of the inflammatory microenvironment.

In the OA joint microenvironment, the localization and function of CD14^+^ cells exhibit significant heterogeneity depending on tissue region, cell subset, and disease stage. At the cellular level, CD14 is definitively expressed in the monocyte/macrophage lineage, defined as a monocyte/macrophage pattern-recognition receptor, and CD14^+^ cell-enriched fractions can be successfully isolated from OA synovial fluid mononuclear cells and synovial tissues (21, 23, 24, 35). In synovial fluid, CD14^+^ monocytes/macrophages account for a median proportion of 47.4% (range 7.1%–94.4%) of CD45^+^ immune cells, and the number of CD14^+^ macrophages in synovial fluid from patients with moderate post-traumatic OA is significantly higher than that in mild OA and controls (36, 37). At the tissue distribution level, CD14^+^ cells are mainly located in the synovium (clearly separable by magnetic-activated cell sorting into CD14^+^ macrophage-rich and CD14^-^ fibroblast-rich fractions) (38) and in synovial fluid (in both membrane-bound and soluble sCD14 forms) (39); CD14^+^ macrophage infiltration is also present in the infrapatellar fat pad (IPFP), with a subset distribution different from that in the synovium (40, 41). In contrast, no experimental evidence supports CD14 expression in chondrocytes, subchondral bone parenchymal cells, or lymphocytes. At the functional heterogeneity level, synovial CD14^+^ macrophages highly express IRF5 and IL-12, and IRF5 levels positively correlate with OA radiographic stage (Stage 4 significantly higher than Stage 2/3) (42). CD14^+^CD16^+^ intermediate monocytes express higher levels of TLR4 than CD14^++^CD16^-^ classical monocytes (23). M1-polarized macrophages markedly upregulate IRF5 and drive the “M1-Th1 inflammatory axis” through an IL-12-dependent mechanism (42). In addition, CD14^+^ synovial tissue macrophages can mediate efferocytosis of apoptotic cells, a process dependent on the interaction between a specific GAPDH isoform exposed on apoptotic cells and membrane CD14 on phagocytes (43, 44). Soluble CD14 (sCD14) levels in synovial fluid are positively correlated with knee hyperalgesia, WOMAC score, and inflammatory markers; recombinant CD14 together with LPS stimulates synovial fibroblast-like cells to release pro-inflammatory cytokines, suggesting that sCD14 participates in synovial stromal–immune cell crosstalk (25). Animal models further confirm that CD14 gene deletion or intra-articular CD14 blockade significantly reduces synovial and IPFP inflammation, pain-related behaviors, and modulates high-dimensional transcriptomic and proteomic inflammatory pathways (25). Collectively, the heterogeneity of CD14 in the OA microenvironment is manifested as multi-dimensional differences in spatial distribution (synovial fluid/synovium/IPFP), cell subsets (CD14^+^CD16^+^ vs CD14^++^CD16^-^ subsets, M1/M2 polarization states), and disease stage (dynamic association with radiographic stage and model severity), providing a cellular and molecular basis for targeted intervention of CD14 pathways.

Furthermore, with the development of single-cell transcriptomics, spatial transcriptomics, and histological techniques, macrophage heterogeneity has been systematically revealed at multiple dimensions. At the anatomical localization level, TIM4^+^CX3CR1^+^ tissue-resident macrophages exist in the synovial sublining, which are independent of monocyte origin, localize near vascularized structures and constitute a unique “synovial niche”; in contrast, monocyte-derived TIM4^-^MHCII^+^ macrophages display a different distribution pattern (45). Immunohistochemistry further confirms significant spatial heterogeneity of CD68^+^ macrophage distribution in the synovial sublining, and lining macrophages, due to their direct contact with joint cavity contents, may participate in sensing synovial fluid factors and initiating immune responses (46). At the transcriptional subset level, single-cell sequencing has identified CD74^+^ macrophages (exhibiting strong pro-inflammatory transcriptional features and considered a key cell population driving synovial inflammation) (47), CXCL16^+^ macrophages (significantly expanded during OA progression, possibly involved in immune cell recruitment) (48), and senescence-associated macrophages (increased in traumatic OA, with their senescence-associated secretory phenotype potentially exacerbating local inflammation and tissue damage) (49). At the polarization level, flow cytometry has definitively identified M1(CD68^+^CD16^+^CD206^-^) and M2 (CD68^+^CD206^+^CD16^-^) phenotypes in OA synovium and synovial fluid; the proportion of pro-inflammatory M1-like macrophages is increased in OA synovium, while anti-inflammatory M2-like macrophages are relatively insufficient, and this imbalance is regarded as a marker of OA progression (50, 51). Interstitial synovial M2 subsets maintain joint homeostasis under physiological conditions and possess the potential to suppress chronic inflammation and promote tissue repair, making them candidate targets for disease-modifying cell-based therapies (52). At the cellular interaction level, TIM4^+^CX3CR1^+^ macrophages interact with synovial fibroblasts through the OSM/OSMR signaling network to drive fibrosis and inflammation (45); fibroblast-derived C3 interacts with macrophage C3aR1 to enhance type I interferon responses (53). At the cross-tissue heterogeneity level, macrophage subset distribution in the IPFP differs from that in the synovium, and its M1/M2 ratio significantly correlates with disease severity. Macrophages account for nearly half of CD45^+^ cells in synovial fluid, and their subset composition is closely associated with T-cell activation status. Based on macrophage transcriptional features, OA can be classified into inflammatory OA and classical OA subtypes, providing a basis for precision stratified therapy (54, 55). In summary, OA synovial macrophages exhibit multi-dimensional heterogeneity in anatomical localization, transcriptional subsets, polarization states, disease subtype associations, and cross-tissue distribution. The integrated application of single-cell and spatial transcriptomic technologies has laid a cellular foundation for understanding the OA immune microenvironment and developing disease-modifying strategies targeting specific subsets.

However, although current evidence on CD14 heterogeneity in the OA microenvironment has preliminarily revealed its spatial distribution and functional diversity, it must be critically appraised at multiple levels. At the causal inference level, the cross-sectional association between synovial fluid sCD14 levels and pain/effusion cannot distinguish whether sCD14 is a consequence of inflammation or a pathogenic driver. Although CD14 gene knockout or intra-articular blockade attenuates inflammation and pain in post-traumatic OA mouse models, these models poorly recapitulate the chronic low-grade inflammatory features of human primary OA; global deletion may disturb non-articular tissue homeostasis, and the target specificity and durability of intra-articular antibody blockade have not been adequately validated (25). At the heterogeneity characterization level, existing data are heavily concentrated on end-stage knee OA synovial fluid and synovium, with hip OA and spinal OA samples lacking. Flow cytometric sorting cannot distinguish tissue-resident macrophages from monocyte-derived macrophages. Although single-cell sequencing reveals immune cell subset diversity, it does not clearly define the transcriptional subsets, developmental trajectories, or chondrocyte interaction networks of CD14^+^ cells. All human samples are obtained from end-stage surgery, failing to reflect the spatiotemporal evolution of CD14^+^ cells during early OA stages (56, 57). Future studies should combine multiplex immunofluorescence or spatial transcriptomics to precisely map CD14^+^ cells in the synovial lining/sublining and around IPFP vessels; distinguish the dual roles of CD14 in pro-inflammatory signaling versus tissue homeostasis maintenance; stratify CD14 expression according to OA etiology, inflammatory phenotypes, and pain phenotypes to match precision therapeutic needs; and utilize humanized mouse models or ex vivo joint culture systems for cross-species validation.

## 3 CD14-mediated signaling mechanisms

**Figure 1:**
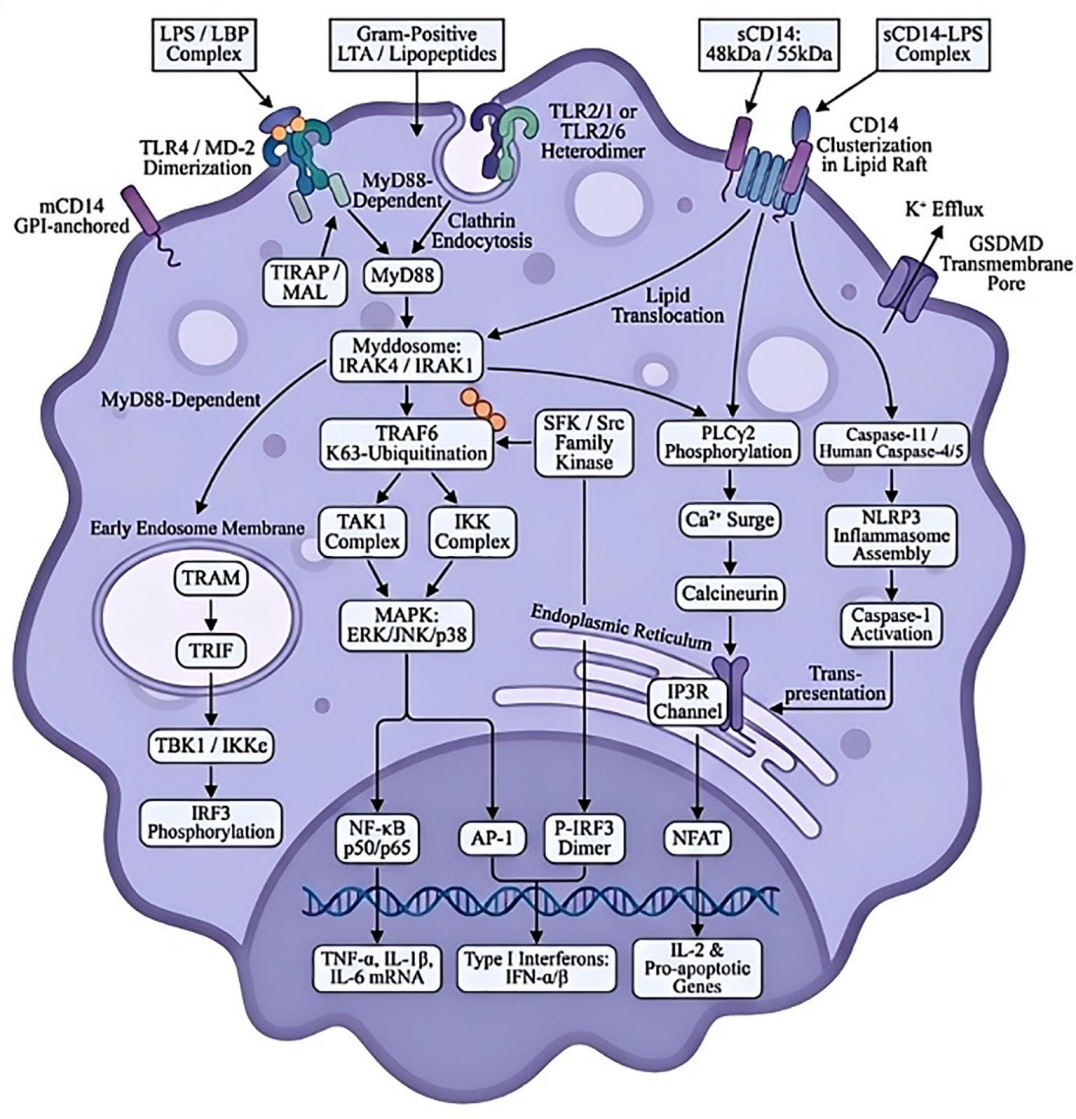
Schematic diagram of the CD14-mediated signaling mechanism.

As a key pattern recognition receptor, CD14 recognizes multiple PAMPs and DAMPs. In the OA microenvironment, CD14 can recognize various endogenous and exogenous ligands, including LPS, high−mobility group box 1 (HMGB1), β−amyloid (Aβ), and apolipoprotein C3 (ApoC3) (58–60). Among these, LPS is the most classical ligand for CD14; upon binding to CD14, it further recruits TLR4 to form the CD14/TLR4 complex, thereby initiating downstream inflammatory signaling (61). In addition, in OA synovial fluid, CD14 can respond to endogenous DAMPs such as HMGB1 and cartilage degradation products to activate innate immune responses (58). Notably, CD14’s recognition of ligands is not limited to TLR-dependent pathways but can also directly regulate inflammasome activation through non-classical mechanisms (60). The diagram of the CD14-mediated signaling mechanism is shown in **Figure 1**.

CD14 plays a central auxiliary role in TLR4−dependent signal transduction. After ligands such as LPS bind to CD14, CD14 transfers LPS to the TLR4−MD2 complex, inducing TLR4 dimerization and initiating two major signaling pathways: the membrane−localized MyD88−dependent pathway and the endosomal TRIF−dependent pathway. The MyD88 pathway rapidly activates NF−κB and MAPK signaling, promoting the transcription of pro−inflammatory cytokines such as IL−6, TNF−α, and pro−IL−1β (23, 30, 62); the TRIF pathway, in turn, activates IRF3 with delayed kinetics, inducing type I interferon production and participating in the “priming” phase of the NLRP3 inflammasome (61). In OA, this pathway has been confirmed to be widely activated: synovial fibroblasts stimulated with LPS significantly upregulate IL−6, IL−8, and matrix metalloproteinase−3 (MMP−3) via the CD14/TLR4/NF−κB axis; simultaneously, elevated TLR4 expression in chondrocytes is closely associated with NLRP3 inflammasome activation (23, 63). Moreover, inhibition of TLR4 expression or function (e.g., using CLI−095) effectively blocks NLRP3 activation and IL−1β release, attenuating cartilage degeneration (59).

In addition to TLR4, CD14 can also cooperate with TLR2 to mediate inflammatory signaling (64). Studies indicate that certain DAMPs can induce the release of IL−6 and MCP−1 in chondrocytes, synoviocytes, and macrophages through TLR2 or TLR4, suggesting that CD14 may serve as a co−receptor in the assembly of the TLR2 signaling complex (62). In a Neisseria gonorrhoeae infection model, TLR2—rather than TLR4—was shown to activate the NLRP3 inflammasome, a process involving NF−κB and MAPK pathway−mediated priming signals as well as activating signals such as potassium efflux and mitochondrial ROS production (65). Although the specific role of TLR2 in OA is less well defined than that of TLR4, soluble TLR2 has been detected in synovial fluid, and its levels differ among OA types of different joints, hinting that the TLR2/CD14 axis may operate in specific OA subtypes (66). Furthermore, ApoC3 can induce TLR2−TLR4 heterodimerization and co−activate the non−canonical NLRP3 inflammasome, further expanding the role of CD14 in TLR2−mediated pathways (60).

Recent studies have uncovered that CD14 can mediate signaling independently of TLR4. On one hand, CD14 can regulate dendritic cell (DC) apoptosis and immune tolerance through activation of the nuclear factor of activated T cells (NFAT) pathway; although this mechanism has not been explicitly verified in OA, it has been reported in other inflammatory models. On the other hand, CD14 can directly participate in non−canonical activation of the NLRP3 inflammasome. For instance, in human monocytes, LPS can bind CD14 and activate caspase−4/5/11 (caspase−11 in mice), which then cleaves gasdermin D and promotes NLRP3 inflammasome assembly, ultimately leading to mature IL−1β release (61). In an intervertebral disc degeneration model, sLN directly interacts with the LPS−binding site of CD14, inhibiting both TLR4−dependent signaling and caspase−1 activation/IL−1β secretion, indicating that CD14 itself can serve as a regulatory node for inflammasome activity (61). Additionally, ApoC3 activates a caspase−8−dependent non−canonical NLRP3 pathway through CD14−mediated calcium influx and ROS generation, a process that is TLR4−independent but requires CD14 (60). These findings suggest that CD14 may directly drive inflammasome activation in OA via TLR4−independent mechanisms.

In OA patient synovial fluid, sCD14 levels are significantly elevated and positively correlate with IL−6, IL−1β, TNF−α, and complement C3 (23). *In vitro* experiments confirmed that recombinant sCD14 together with LPS synergistically promotes the secretion of IL−6, IL−8, and MMP−3 by OA fibroblast−like synoviocytes (23). In addition, sCD14 levels correlate with knee hyperalgesia and joint effusion volume, and CD14 deficiency or local blockade alleviates synovial inflammation and pain behaviors in mice. sCD14 not only serves as a carrier for LPS but also recognizes endogenous DAMPs and can initiate TLR4 signaling in cells lacking mCD14, thereby expanding the scope of inflammatory responses (25). Therefore, sCD14 in the OA joint microenvironment acts both as an inflammatory amplifier and a potential systemic inflammatory indicator.

The CD14−mediated signaling network is highly complex, involving cross−talk among TLR4−dependent, TLR2−dependent, and TLR−independent pathways. In OA, CD14 coordinates NF−κB priming signals and NLRP3 inflammasome activation signals, forming a “two−signal” cascade that amplifies inflammation (61, 63). For example, LPS via CD14/TLR4 activates NF−κB to upregulate NLRP3 and pro−IL−1β expression (priming phase), and then secondary signals such as ATP or crystals trigger NLRP3 assembly and caspase−1 activation (activation phase) (67). Furthermore, CD14 interacts with other regulatory molecules: YBX1 can directly bind TLR4 and inhibit its signaling (68); icariin (ICA) attenuates NLRP3 activation by suppressing the TLR4/P2rx7/NF−κB pathway (69); and nicardipine blocks NLRP3 priming by inhibiting TLR4 expression and NF−κB activation (70). These regulatory nodes suggest that CD14 does not act in isolation but is embedded in a dynamically balanced signaling network. In OA progression, the imbalance of this network leads to persistent low−grade inflammation, driving cartilage degradation and synovial hyperplasia.

## 4 Role of CD14 in the pathological process of osteoarthritis

Numerous studies indicate that CD14 exerts a significant pro−inflammatory role in OA inflammation. In mouse joint injury−induced OA models, CD14 gene deficiency effectively alleviates structural lesions such as cartilage damage and subchondral bone remodeling, and improves motor dysfunction, suggesting that CD14 participates in driving OA pathological progression (24). Clinical sample analyses further support this view: CD14 expression is significantly elevated in OA synovial tissue, and its positive areas (mainly macrophage−rich regions) show markedly higher TNF−α and IL−1β mRNA levels than CD14−negative areas (fibroblast−rich regions) (20). *In vitro* experiments show that recombinant CD14 combined with LPS synergistically promotes the release of IL−6, IL−8, and MMP−3 from OA fibroblast−like synoviocytes, exacerbating synovial inflammation and matrix degradation (23). Moreover, sCD14 levels in synovial fluid are significantly positively correlated with concentrations of IL−6, IL−1β, TNF−α, and complement C3, and are associated with joint effusion volume and knee hyperalgesia. Notably, in multiple post−traumatic OA mouse models (covering different severities, sexes, and obesity statuses), both systemic CD14 deficiency and local CD14 blockade significantly reduce synovial and infrapatellar fat pad inflammation, alleviate pain behaviors, and improve motor capacity, further confirming the central pro−inflammatory role of CD14 in OA inflammatory regulation (25). These lines of evidence collectively demonstrate that CD14 acts as a key driving factor in the chronic low−grade inflammatory environment of OA by activating pro−inflammatory signaling pathways, promoting the release of inflammatory cytokines, and amplifying synovial immune responses. Recent evidence supporting CD14 involvement in OA is shown in **Table 1**.

**Table 1:** Evidence supporting the involvement of CD14 in OA.

Study	Sample	OA-specific?	Key findings	Level of evidence
Human cross-sectional clinical study (23, 25)	Synovial fluid from 35 knee OA patients (9 males, 26 females; mean age 66.3 ± 8.8 years)	focused on the knee OA; correlated with clinical symptoms	Soluble CD14 (sCD14) levels in synovial fluid were significantly positively correlated with knee hyperalgesia severity, effusion volume, and WOMAC scores (progression indicators).	Moderate (Observational human study providing clinical correlation but cannot establish causality)
Multi-model *in vivo* intervention study (24, 25)	0-week-old male C57BL/6 wild-type vs. CD14^-^/^-^ mice; Multiple post-traumatic OA (PTOA) surgical models (mild to severe pathology); intra-articular anti-CD14 antibody delivery	models specifically designed for OA (joint injury-induced); no RA or other inflammatory arthritis controls	CD14 deficiency significantly reduced synovitis, pain-related behaviours, and histopathological cartilage damage. Intra-articular anti-CD14 antibody alleviated synovial and infrapatellar fat pad inflammation. High-dimensional transcriptomic and proteomic analyses confirmed CD14-dependent regulation of synovial inflammation pathways.	High (Multi-model, multi-endpoint functional studies in animals providing causal mechanistic evidence)
Human synovial cell subset functional analysis (23, 35)	Synovial tissue from knee OA and rheumatoid arthritis (RA) patients; CD14^+^ vs. CD14^-^ subsets isolated by magnetic bead/flow sorting	CD14^+^ cells are enriched in OA synovium; OA vs. RA comparison revealed unique inflammatory features	MINCLE expression was significantly higher in CD14^+^ vs. CD14^-^ fractions (in both OA and RA). In OA, CD14^+^ Monocytes/macrophages serve as key effector cells of synovial inflammation. The CD14^-^ fraction showed higher MINCLE in RA than OA, suggesting OA-specific CD14^+^ cell inflammation characteristics.	Moderate–High (Direct human tissue evidence with cross-disease comparison, reinforcing the pathological role of CD14^+^ cells in OA)
*In vitro* mechanistic study *(*25)	Fibroblast-like synoviocytes (FLSs) from OA patients; recombinant CD14 + LPS co-stimulation	cells derived from OA patients, but stimulus model represents a general inflammatory pathway	CD14 participates in inducing the release of inflammatory cytokines (e.g., IL-6, IL-8, MMP-3) from synovial stromal cells, suggesting crosstalk between CD14^+^ myeloid cells and synovial tissue.	Moderate (*in vitro* mechanistic support, requiring *in vivo* validation)

Beyond its pro−inflammatory functions, CD14 has also been found to participate in the regulation of apoptotic cell clearance (efferocytosis), a process critical for maintaining joint immune homeostasis. Studies have revealed that a specific conformation of GAPDH exposed on apoptotic cell surfaces can directly bind to CD14 on phagocyte membranes, mediating efferocytosis, and establishing for the first time a novel function of CD14 as a phagocytic receptor (44, 71, 72). In human monocytes/macrophages, CD14 cooperates with MerTK; antibody−mediated cross−linking of CD14 activates Syk kinase and induces MerTK phosphorylation, thereby enhancing the phagocytic capacity of M2c macrophages toward apoptotic neutrophils; simultaneously, CD14 and MerTK are co−expressed on M2c microvesicles, suggesting that they form functional complexes in specific macrophage subsets to promote efferocytosis (73). In the OA pathological context, the efferocytosis function of synovial macrophages is generally impaired, leading to accumulation of apoptotic cells and secondary release of inflammatory mediators such as TNF−α, IL−1β, and IL−6, which exacerbate synovitis and cartilage destruction. Notably, TGM2 can influence macrophage scavenger function by regulating the expression levels of CD14 and SR−AI receptors; inhibition of TGM2 leads to decreased CD14 expression and promotes a pro−inflammatory phenotype, indirectly suggesting the necessity of CD14 in maintaining macrophage phagocytic capacity (74). In addition, some studies have used macrophage−targeted immunoliposomes to activate molecules such as PROS1 and TIMD4, enhancing efferocytosis capacity of M0 macrophages and promoting their polarization toward an anti−inflammatory M2 phenotype, thereby suppressing inflammation and facilitating cartilage repair (75); although CD14 was not directly examined in that study, given its role in macrophage recognition of apoptotic cells, it may participate in this regulatory network. Collectively, CD14 may exert immunomodulatory functions in OA through mediating efferocytosis, preventing secondary necrosis of apoptotic cells and the ensuing inflammatory cascade.

Most evidence points to the pro−inflammatory actions of CD14, and whether it has potential protective functions in OA remains controversial. On one hand, none of the studies have shown that CD14 itself, as a receptor, directly recognizes apoptotic cells or mediates efferocytic signaling; in efferocytosis research, CD14 is used only as a cell marker, and its functional role is limited to identifying macrophage populations that perform efferocytosis (52). CD14 deficiency or blockade alleviates OA synovial inflammation and pain, but these studies did not examine whether such intervention concurrently improves efferocytosis function (25). The benefit of CD14 inhibition is more likely derived from blocking the TLR4/NF−κB pro−inflammatory pathway rather than directly correcting efferocytosis defects (24). On the other hand, current studies demonstrate that CD14 is widely expressed on monocytes/macrophages, certain dendritic cell subsets, neutrophils, and some non−immune cells (e.g., vascular smooth muscle cells, tumor cells) (25, 32, 76, 77). However, within the OA microenvironment, the cells that definitively express CD14 include synovial macrophages (core), monocytes (enriched in synovial fluid), chondrocytes, synovial fibroblasts, and myeloid cells in the infrapatellar fat pad, and their functions are focused on pro−inflammatory effects: (i) driving synovial inflammation (via the TNF−α/IL−1β/NGF axis); (ii) mediating pain signals; and (iii) promoting cartilage and bone structural destruction (23, 32).

## 5 Targeted intervention of CD14 in osteoarthritis

The anti−CD14 monoclonal antibody represented by IC14 (atibuclimab) is the current major therapeutic tool targeting this pathway (78, 79). IC14 has entered human clinical trials; although its humanized engineering characteristics are not detailed in the available studies, based on its Phase I trial and expanded access protocol application in patients with amyotrophic lateral sclerosis, it is reasonable to infer that it is designed to reduce immunogenicity (80). At the mechanistic level, existing evidence supports that such antibodies likely target mCD14, sterically hindering the formation of the LPS/TLR4 signaling complex and thereby inhibiting downstream NF−κB−mediated pro−inflammatory responses (25, 81). In the OA field, intra−articular blockade of CD14 has gained solid preclinical validation. Multiple independent studies in post−traumatic OA mouse models have confirmed that intra−articular injection of CD14−neutralizing antibody significantly reduces synovial inflammation and histopathological damage, and effectively alleviates pain behaviors such as mechanical hyperalgesia, with effects consistently reproduced across various models and both sexes (25, 80). The core mechanism lies in suppressing the release of key pro−inflammatory cytokines such as TNF−α, IL−1β, and IL−6 from synovial CD14^+^ macrophages, thus breaking the “inflammation–pain–cartilage degeneration” vicious cycle. The positive correlation between human OA synovial fluid sCD14 levels and joint effusion and hyperalgesia further supports CD14 as a clinically relevant disease−modifying target (25).

Cross−disease references provide translational support for OA application. In lethal sepsis models, anti−CD14 antibody significantly inhibits coagulation dysfunction and organ damage (82); protective effects have also been shown in renal and hepatic ischemia−reperfusion injury models (83, 84). The safety data of IC14 in ALS patients provide preliminary human evidence for local administration strategies (78). It should be noted that current evidence has not reported clinical trial results of CD14−blocking antibodies in OA patients, nor has it clearly distinguished antibody selectivity for mCD14 versus sCD14. Overall, the available literature indicates that local CD14 blockade has a reasonable scientific basis for translation from preclinical to clinical settings, but systematic evaluation of intra−articular pharmacokinetics, long−term safety, and patient stratification strategies is still needed to provide clear direction for subsequent study design.

In addition, novel targeting strategies are expanding from single−antibody blockade toward cell engineering and combination interventions. In terms of delivery and gene editing, nanocarriers can achieve precise drug delivery to CD14^+^ macrophages to reduce off−target toxicity, and CRISPR−Cas9 has been successfully delivered to primary human CD14^+^ monocytes for efficient gene editing; combined with the mechanistic validation of global CD14 knockout in OA models, future exploration may target CD14 or TLR4 editing specifically in CD14^+^ macrophages for cell−specific intervention (21, 85, 86). In cell engineering, current CAR−T manufacturing protocols require removal of CD14^+^ cells to avoid interference with T−cell expansion; although it is theoretically possible to design CD14−targeted CAR−T to eliminate pathogenic macrophages in the joint, given that CD14^+^ cells are considered “contaminating” components and OA is a chronic degenerative disease, off−target toxicity must be carefully considered, and tumor experience should not be directly extrapolated (87, 88). Bispecific antibody strategies leverage mature technologies such as knob−into−hole; since combined CD14 and C5 blockade can maximize suppression of cytokine release, bispecific antibodies targeting CD14 together with the complement pathway (e.g., C5) have synergistic anti−inflammatory potential and align with the positioning of monoclonal antibodies as disease−modifying OA drugs (89, 90). In combination therapy, based on the pathological evidence that CD3^+^ T cells dominate synovial lymphocytes and that Treg/Th17 imbalance exists, CD14 blockade (suppressing macrophage−derived TNF−α/IL−1β) combined with T−cell checkpoints (such as PD−1/LAG−3) might break the “macrophage–T cell” inflammatory crosstalk, but no direct experimental data are available in the literature (21). Meanwhile, drawing on evidence that TAA treatment reduces the proportion of pro−inflammatory CD14^+^/CD80^+^ and CD14^+^/CD86^+^ macrophages while increasing anti−inflammatory CD14^+^/CD163^+^ macrophages, CD14 blockade to reduce inflammation could be followed by combined injection of IL−4/IL−10 or MSCs to promote an “anti−inflammatory + pro−repair” sequential therapy (25, 91). Furthermore, based on the function of Tregs in suppressing inflammation and promoting bone repair, and the observation that Tregs co−cultured with CD14^+^ monocytes increase the proportion of CD206^+^CD14^+^ M2−like monocytes, CD14 blockade followed by CAR−Treg infusion may synergistically promote inflammation resolution and tissue repair (92). Nanodelivery, CRISPR technology, and cell engineering have provided a diversified toolkit and theoretical foundation for CD14-targeted interventions. However, therapeutic studies of CD14 in OA remain characterized by “well-established target validation, limited direct intervention strategies, and cell engineering applications that have not yet been translated to clinical practice.” All extensions must be rigorously validated for local safety and long-term effects. Moreover, clinical data on CAR-T targeting CD14 and bispecific antibodies are still absent, and related hypotheses urgently require experimental evidence for support.

## 6 Conclusions and perspectives

Collectively, the available evidence indicates that CD14 primarily exerts a clear pro−inflammatory driving role in OA. Clinical samples and animal models consistently show that CD14 is highly expressed on OA synovial macrophages, and its levels positively correlate with inflammatory cytokines (IL−6, IL−1β, TNF−α) as well as clinical symptoms such as pain and joint effusion; CD14 gene deficiency or local antibody blockade significantly reduces cartilage damage, synovial inflammation, and pain behaviors. Although some studies suggest that CD14 may participate in apoptotic cell clearance (efferocytosis) or immune tolerance regulation, there is currently no direct evidence that these functions are sufficient to counteract its pro−inflammatory effects in the OA microenvironment, and the “protective” role of CD14 in OA still requires more direct experimental verification.

Significant gaps remain in current research: first, the differential functions and signaling efficiencies of mCD14 versus sCD14 in OA have not been systematically distinguished, and the release mechanisms of sCD14 and its pathogenic contribution in mCD14−negative cells such as fibroblast−like synoviocytes remain unclear; second, the functional heterogeneity of CD14 among different immune cell subsets (e.g., classical CD14^+^CD16^-^ monocytes vs. intermediate CD14^++^CD16^+^ monocytes) is underexplored; third, whether CD14 regulates macrophage polarization through metabolic reprogramming, and its role in metabolic inflammation in obesity−associated OA, are still unknown.

Looking forward, single−cell transcriptomics and spatial transcriptomics are expected to resolve the heterogeneity of CD14^+^ myeloid cells at the single−cell level and their spatial distribution and functional subtypes within the synovial microenvironment, providing a cellular basis for precision targeting. In terms of therapeutic strategies, priority should be given to developing highly selective CD14−blocking antibodies or small−molecule inhibitors, and exploring intra−articular sustained−release delivery systems to improve local safety; simultaneously, patient stratification using fluid biomarkers such as sCD14 should be pursued to facilitate biomarker−driven enrichment clinical trial designs. In addition, the potential impact of systemic CD14 inhibition on host defense must be monitored; future interventions should aim at selectively blocking its pro−inflammatory signaling branches rather than completely abrogating CD14 function, in order to achieve a balance between precision and safety.
